# Patient, Family, and Clinician Perspectives on End-of-Life Care Quality Domains and Candidate Indicators for Adolescents and Young Adults With Cancer

**DOI:** 10.1001/jamanetworkopen.2021.21888

**Published:** 2021-08-23

**Authors:** Jennifer W. Mack, Lauren Fisher, Larry Kushi, Chun R. Chao, Brenda Vega, Gilda Rodrigues, Isabel Josephs, Katharine E. Brock, Susan Buchanan, Mallory Casperson, Robert M. Cooper, Karen M. Fasciano, Tatjana Kolevska, Joshua R. Lakin, Anna Lefebvre, Corey M. Schwartz, Dov M. Shalman, Catherine B. Wall, Lori Wiener, Andrea Altschuler

**Affiliations:** 1 Division of Population Sciences, Dana-Farber Cancer Institute, Boston, Massachusetts; 2 Department of Pediatric Oncology, Dana-Farber Cancer Institute, Boston, Massachusetts; 3 Division of Research, Kaiser Permanente Northern California, Oakland; 4 Department of Research and Evaluation, Kaiser Permanente Southern California, Pasadena; 5 Brown Medical School, Providence, Rhode Island; 6 Divisions of Pediatric Oncology and Palliative Care, Emory University and Aflac Cancer & Blood Disorders Center at Children’s Healthcare of Atlanta, Atlanta, Georgia; 7 Department of Medical Oncology, Dana-Farber Cancer Institute, Boston, Massachusetts; 8 Now with Agios Pharmaceuticals, Cambridge, Massachusetts; 9 Lacuna Loft, Oakland, California; 10 Department of Pediatric Oncology, Kaiser Permanente Southern California, Pasadena; 11 Department of Psychosocial Oncology and Palliative Care, Dana-Farber Cancer Institute, Boston, Massachusetts; 12 Division of Medical Oncology, Kaiser Permanente Northern California, Oakland; 13 Department of Palliative Care, Kaiser Permanente Southern California, Pasadena; 14 Psychosocial Support and Research Program, National Cancer Institute, Bethesda, Maryland

## Abstract

**Importance:**

End-of-life care quality indicators specific to adolescents and young adults (AYAs) aged 12 to 39 years with cancer have not been developed.

**Objective:**

To identify priority domains for end-of-life care from the perspectives of AYAs, family caregivers, and clinicians, and to propose candidate quality indicators reflecting priorities.

**Design, Setting, and Participants:**

This qualitative study was conducted from December 6, 2018, to January 5, 2021, with no additional follow-up. In-depth interviews were conducted with patients, family caregivers, and clinicians and included a content analysis of resulting transcripts. A multidisciplinary advisory group translated priorities into proposed quality indicators. Interviews were conducted at the Dana-Farber Cancer Institute, Kaiser Permanente Northern California, Kaiser Permanente Southern California, and an AYA cancer support community (lacunaloft.org). Participants included 23 AYAs, 28 caregivers, and 29 clinicians.

**Exposure:**

Stage IV or recurrent cancer.

**Main Outcomes and Measures:**

Care priorities.

**Results:**

Interviews were conducted with 23 patients (mean [SD] age, 29.3 [7.3] years; 12 men [52%]; 18 White participants [78%]), 28 family caregivers (23 women [82%]; 14 White participants [50%]), and 29 clinicians (20 women [69%]; 13 White participants [45%]). Caregivers included 22 parents (79%), 5 spouses or partners (18%), and 1 other family member (4%); the 29 clinicians included 15 physicians (52%), 6 nurses or nurse practitioners (21%), and 8 social workers or psychologists (28%). Interviews identified 7 end-of-life priority domains: attention to physical symptoms, attention to quality of life, psychosocial and spiritual care, communication and decision-making, relationships with clinicians, care and treatment, and independence. Themes were consistent across the AYA age range and participant type. Although some domains were represented in quality indicators developed for adults, unique domains were identified, as well as AYA-specific manifestations of existing domains. For example, quality of life included global quality of life; attainment of life goals, legacy, and meaning; support of personal relationships; and normalcy. Within communication and decision-making, domains included communication early in the disease course, addressing prognosis and what to expect at the end of life, and opportunity for AYAs to hold desired roles in decision-making. Care and treatment domains relevant to cancer therapy, use of life-prolonging measures, and location of death emphasized the need for preference sensitivity rather than a standard path. This finding differs from existing adult indicators that propose that late-life chemotherapy, intensive measures, and hospital death should be rare.

**Conclusions and Relevance:**

The findings of this qualitative study suggest that AYAs with cancer have priorities for care at the end of life that are not fully encompassed in existing indicators for adults. Use of new indicators for this young population may better reflect patient- and family-centered experiences of quality care.

## Key Points

**Question:**

What do adolescents and young adults with cancer, their families, and clinicians find important in end-of-life care?

**Findings:**

This qualitative study of interviews with 23 adolescents and young adults, 28 family caregivers, and 29 clinicians identified 7 distinct end-of-life priority domains: attention to physical symptoms, attention to quality of life, psychosocial and spiritual care, communication and decision-making, relationships with clinicians, care and treatment, and independence. Although some domains reflected existing quality domains for adults, some domains were unique or had distinct manifestations for this young population.

**Meaning:**

The priority domains identified in this study as relevant to adolescents and young adults with advanced cancer may be used for quality measurement.

## Teaser

This qualitative study assesses patient, family, and clinician perspectives on priority domains for end-of-life care for adolescents and young adults with cancer.

## Introduction

More than 70 000 adolescent and young adult (AYA) patients with cancer are diagnosed in the US each year, and cancer is their leading disease-related cause of death.^1,2^ This population is at risk for inferior quality cancer care,^1,3,4,5,6,7,8,9,10,11,12,13,14,15^ in part because AYAs are at transitional phases in life and thus may have unique social, educational, employment, and family concerns beyond their treatment needs.^4,5^

Adolescents and young adults may be especially vulnerable to care challenges as they face death. These deaths are a departure from the natural order of life in which each generation outlives the one before it.^16^ In contrast to feelings that their lives should be ahead of them, dying young people face profound losses, including the opportunity to find partners, nurture families and careers, and grow old.^16,17^ Adolescents and young adults near death also often recognize the pain they leave behind in surviving loved ones.^18^ All of these issues may contribute to heightened use of intensive measures at the end of life in this population.^19,20^

To date, end-of-life care quality indicators specific to AYAs with cancer have not been developed, challenging our ability to ascertain and measure optimal care for AYAs approaching death. Existing quality indicators for adults with cancer have at times been used to understand end-of-life care among AYAs and have identified high rates of inpatient care, care in the intensive care unit or emergency department, and chemotherapy near death.^19,20^ However, we do not know whether these indicators, and other indicators developed for adults, speak to the values of AYAs. In addition, existing quality indicators for adults often take a health care systems perspective, using health care utilization data, with a focus on management of pain and symptoms,^21,22,23,24,25,26,27^ limited use of intensive measures,^23,25,26,28,29^ and involvement of palliative care and hospice.^21,23,25,26,27,30,31^ Although health care systems–based indicators are important, they do not speak to the individual patient and family experience of care. The 2013 National Academy of Medicine report, *Delivering High-Quality Cancer Care: Charting a New Course for a System in Crisis*,^32^ recommends use of an array of indicators defined from multiple perspectives, including process indicators based on care delivery and indicators that evaluate the patient and family experience, noting “pervasive gaps in existing cancer measures”^32^^(p15)^ due to “a lack of consumer engagement in measure development.”^32^^(p15)^

The goal of this study was to develop a patient-centered definition of high-quality end-of-life care for AYAs with cancer and to use that definition to propose quality domains and indicators. We conducted qualitative interviews with AYA patients aged 12 to 39 years with advanced (stage IV or recurrent) cancer; family caregivers of living AYAs with advanced cancer, as well as bereaved family caregivers; and multidisciplinary clinicians who care for AYAs with advanced cancer. Interviews were designed to identify domains of importance to AYAs and their family members. We then worked with a 12-member multidisciplinary advisory group, including physicians in medical and pediatric oncology and hospice or palliative medicine, pediatric oncology nurse practitioners, a medical oncology physician assistant, a patient advocate, and psychosocial health care professionals, to translate priority domains into proposed quality indicators. When possible, existing quality indicators were recommended, but in select areas, a need for new indicators was identified when existing quality indicators were not adequate to speak to identified domains.

## Methods

Participants were recruited for this qualitative study between December 6, 2018, and January 5, 2021, from 3 sites (the Dana-Farber Cancer Institute in Boston, Massachusetts; Kaiser Permanente Northern California in Oakland; and Kaiser Permanente Southern California in Pasadena) and online via an AYA cancer support and advocacy organization, Lacuna Loft (lacunaloft.org). Eligible participants included English- or Spanish-speaking AYA patients aged 12 to 39 years with advanced (stage IV or recurrent) cancer; family caregivers of living AYAs with advanced cancer, or of AYAs with cancer who had died within the past 5 years; and clinicians (physicians, nurses or nurse practitioners, and psychosocial clinicians) who care for AYAs with advanced cancer. The age range of 12 to 39 years was chosen to cover both the World Health Organization definition (12-24 years) and National Cancer Institute definition (15-39 years) of an AYA. Eligible patients and caregivers were identified via clinic lists, administrative databases, and/or referrals by clinicians. We used purposive sampling to ensure a range of ages and racial/ethnic representation. An online advertisement on the Lacuna Loft website allowed for self-referral of patients and caregivers. Eligible clinicians were identified by investigators at each site. Signed informed consent was obtained from all participants.

The institutional review boards of the Dana-Farber Cancer Institute and Kaiser Permanente Northern and Southern California approved the study. This report followed the Consolidated Criteria for Reporting Qualitative Research (COREQ) reporting guideline for qualitative research.^33^

With the exception of those who self-referred through Lacuna Loft, permission from the patient’s oncologist was requested for contact of patients and family members. Initial contact was from investigators at the patient’s primary site. Eligible patients (or parents/guardians if younger than 18 years) were approached in person or by mail with a letter describing the study and an informed consent document. Interested patients were asked to respond to a 3-statement readiness assessment to evaluate readiness to discuss end-of-life issues^34^: (1) it might be helpful for me to talk about what would be important to me if treatments were no longer effective; (2) talking about what might be important to me if treatment options were limited or there were no more treatment options available would upset me very much; and (3) I feel comfortable discussing what might be important to me if treatments were no longer effective. Eligible participants were those who responded *yes*, *no*, and *yes*, respectively.

We sought to recruit approximately 20 to 30 patients, 20 to 30 caregivers, and 20 to 30 clinicians, with recruitment through thematic saturation in each group and with representation across the AYA age range, race/ethnicity, caregiver relationship, and clinician type. For patients in the younger age group (12-24 years), we recruited patients and conducted interviews in person to ensure adequate detection of distress. When in-person recruitment was affected by the COVID-19 pandemic, we increased sampling of caregivers of younger patients to enhance representation of their perspectives, recognizing the limitations of caregivers to directly speak to patient perspectives. Sixty individuals declined participation, including 32 patients (or caregivers of adolescent patients who declined on their behalf), 8 caregivers, and 20 clinicians.

Semistructured interviews were conducted at the Dana-Farber Cancer Institute in person or by phone by trained interviewers (J.W.M., B.V., G.R., and I.J.). No interviewers had preexisting relationships with participants; one interviewer, J.W.M., is a pediatric oncologist and conducted limited interviews with medical oncology patients during interviewer training but was not involved in their care. All other interviewers were research assistants. The advisory group reviewed the interview guide to ensure developmental appropriateness and sensitivity. Interviews centered on prognostic awareness; experiences with care; care priorities ; use of medical interventions; differences across the AYA age and developmental spectrum; and nonmedical priorities, such as ways to cultivate meaning (eTable in the Supplement). Patients who disclosed that they had a poor prognosis were asked about personal priorities for care. Those who did not express awareness of poor prognosis were asked to imagine what might be important to them if treatment was no longer working. Caregivers were asked to describe what they felt was most important in the care of their AYA family member, and clinicians were asked to reflect on priorities identified through general experience with AYAs as well as experiences with a recently cared-for AYA with advanced cancer. Interviewers were trained to recognize distress and respond by offering to end the interview early and/or refer the participant for psychosocial support. Some participants, especially bereaved caregivers, expressed grief and sadness; no participants wished to end the interview early or speak with a psychosocial professional.

Interviews were audiorecorded and transcribed. Limited field notes on prognostic awareness and emotional distress were recorded by interviewers. Median interview duration was 31 minutes (range, 14-79 minutes). Transcripts were reviewed for interview quality by J.W.M., with feedback and opportunity for retraining of interviewers if needed. Interviews were offered in English and Spanish, although no participants elected to participate in Spanish. Participation ended once the interview was complete, with no additional follow-up.

### Analysis

Directed content analysis was used to evaluate transcribed interview data^35,36,37,38,39^ using NVivo software, version 1.4 (QSR International). An initial coding schema was developed based on broad domains of high-quality end-of-life care from the literature as well as a priori conceptual categories (eg, attainment of life goals, legacy, support of relationships). Two coders (L.F. and J.W.M.) performed coding independently and then reviewed together, with discrepancies used as opportunities to clarify or expand the coding scheme. New themes were added as they emerged from texts. Coders identified core themes expressed across all interviews, and themes that differed by age, stage in development, and stakeholder (ie, patient, caregiver, or clinician). Participant checking, ie, where interview participants are asked to review study results and comment on them, was not used. The advisory group reviewed results, including select quotations from transcripts, to create an expansive list of end-of-life care priorities.

In a series of virtual meetings, the advisory group then translated care priorities into potential quality indicators, considering ways that high-quality care might support identified priorities. For example, for the domain of attainment of life goals, actual attainment of life goals might not always reflect the quality of care, because not all goals are achievable or a result of efforts by the care team. However, high-quality care systems might ensure recognition of goals and discussion of life goals as part of care planning conversations.

Once potential indicators were identified, we reviewed existing end-of-life care quality indicators within the group, with an eye toward using existing quality indicators when possible. We then delineated areas where new indicators were needed to speak to identified domains and worked within the advisory group to develop those indicators. The advisory group conducted iterative discussions, with drafting and revision of proposed quality indicators, until consensus was obtained, defined as agreement within the group that the proposed indicators were adequate and no new proposals of indicators were obtained.

## Results

Interviews were conducted with 23 patients (mean [SD] age, 29.3 [7.3] years; 12 men [52%] and 11 women [48%]; 18 White participants [78%]), 28 family caregivers (23 women [82%] and 5 men [18%]; 14 White participants [50%]), and 29 clinicians (20 women [69%] and 9 men [31%]; 13 White participants [45%]). Caregivers included 22 parents (79%), 5 spouses or partners (18%), and 1 other family member (4%); the 29 clinicians included 15 physicians (52%), 6 nurses or nurse practitioners (21%), and 8 social workers or psychologists (28%) (Table 1).

**Table 1.** Participant Characteristics

Characteristic	No. (%)
Participating patients	23 (100)
Sex	
Male	12 (52)
Female	11 (48)
Age group, y	
12-24	5 (22)
25-39	18 (78)
Race/ethnicity	
White	18 (78)
Black	0
Hispanic	4 (17)
Asian	0
Other or unknown	1 (4)
Participating family caregivers	28 (100)
Sex	
Male	5 (18)
Female	23 (82)
Race/ethnicity	
White	14 (50)
Black	4 (14)
Hispanic	2 (7)
Asian	0 (0)
Other or unknown	8 (29)
Patient vital status at time of interview	
Living	5 (18)
Deceased	23 (82)
Patient age group, y	
12-24	14 (50)
25-39	14 (50)
Relationship to patient	
Parent	22 (79)
Spouse or partner	5 (18)
Other	1 (3)
Participating clinicians	29 (100)
Sex	
Male	9 (31)
Female	20 (69)
Race/ethnicity	
White	13 (45)
Black	3 (10)
Hispanic	5 (17)
Asian	6 (21)
Other or unknown	2 (7)
Discipline	
Physician	15 (52)
Nurse or nurse practitioner	6 (21)
Social worker or psychologist	8 (27)
Specialty	
Pediatric oncology	5 (17)
Medical oncology	8 (28)
Hospice or palliative medicine	8 (28)
Psychosocial care	8 (28)

We identified 7 distinct end-of-life priority domains across all interviews: attention to physical symptoms, attention to quality of life, psychosocial and spiritual care, communication and decision-making, relationships with clinicians, care and treatment, and independence. No major differences were identified by participant type or across the AYA age range, although bereaved caregivers offered slightly greater focus on the days and hours before death across domains. Domains are shown in Table 2 along with illustrative quotations from participants.

**Table 2.** Quality Domains and Subdomains With Relevant Interview Excerpts

Domains and subdomains	Definition	Interview excerpts^a^
**Attention to physical symptoms**		
Attention to comfort; freedom from symptoms and suffering	Physical and emotional comfort, with as few symptoms and as little suffering as possible	Patient: And for me to feel comfortable most of all... be comfortable.
Cognitive awareness	Opportunity to remain cognitively aware when possible	Caregiver: Whatever would help her with the pain side of things, while balancing that with not being out of it the whole time.
**Attention to quality of life**		
Global quality of life	Overall quality of life as defined by the individual patient	Caregiver: Whatever days he had left, we wanted them to be as enjoyable as possible.
Attainment of life goals, legacy, and meaning	Opportunity to achieve life goals, legacy beyond life, and meaning in life	Patient: Leaving something behind.
		Caregiver: He had made a bucket list and he had shared it with me.
Personal relationships	Support of loved ones and opportunity to maintain or deepen important relationships	Patient: If it all possible, I would like to be around friends and family… So, in that sense, the priority would be—what can I do to make sure that family is taken care of, however I can do that… But there are some family members, where the thing that would best support them for the future would just be conversations, emotional support. I feel like things need to be said that could really make them in a better position to grow and kind of get over some stuff.
Maintaining a sense of normalcy	Continuing aspects of life that allow the patient to feel normal, and being treated as a normal person	Caregiver: No question that what was most important to him was to go to school and be 13. We lived as normal of a life as possible… There’s no question that that was his priority, and that’s what made him happy.
**Psychosocial and spiritual care**		
Spiritual support	Support of spiritual and religious needs	Caregiver: He actively sought out spiritual teachers of various kinds… And he was interested in what was coming to him spiritually, what he was learning as he was making this transition out of life.
Global psychosocial support	Support of psychosocial needs	Clinician: I think the biggest limitation that I've noticed when there is a major struggle is a lot of the spiritual or psychosocial, you know existential suffering that patients are going through as they're coming to terms with the prognosis of what they have. That I feel is usually the biggest barrier that I see in terms of being able to have a better acceptance and understanding of what's happening to them.
**Communication and decision-making**		
Communication about prognosis	Communication about likelihood of cure or length of life	Patient: What’s a likely scenario for me. Like, how much more time do I have? And how well am I doing?
		Caregiver: I was not going to tell him that he is going to pass away, and I'm definitely not going to tell his sister in hopes that she could keep that information to herself. But what bothered me so much is the daily pressure to tell him, like every day. Let me tell you something—one thing I know is my children, and I know that regardless, [sister] will 100 percent understand why we didn't share that information with her. She needed to enjoy her brother just like he needed to enjoy those few days that we had left.
Communication about what to expect at the end of life	Communication about what may happen physically or emotionally as illness progresses and death nears	Caregiver: [She] was really scared… about what was going to happen at the end, what the process was going to be like, that it was going to be unbearable... And so months before, I started asking everyone that I came in contact with—tell me, tell me what this is going to be like. And then… when we got to the point where it seemed important that I conveyed these things to [patient], I mean it didn't set [her] mind completely at ease, but I think it helped some.
Timely communication	Communication about advanced cancer and end of life on a timeline that is right for the patient and family	Caregiver: So, I think if we had talked about [it] at the very beginning, it would have made the end easier because we all would have been clear on what she wanted.
Holding desired role in decision-making	Holding desired level of involvement in decisions about care	Patient: I don't want the decisions to be made for me without me.
		Caregiver: She implicitly trusted us. She knew that we had it, which left her freer to be with her friends and be out from underneath the family roof whenever she could.
		Clinician: If you can get into a conversation with them by themselves—they often do have priorities that don’t align with their parents… I think a lot of teenagers often would be willing to say that they're done trying and fighting so hard earlier, except they either are scared to hurt their parents or the parents just don’t allow that and/or we don’t actually ever get to ask them.
**Relationships with clinicians**		
Therapeutic alliance with clinicians	Sense of connection with clinicians	Caregiver: They talked with him about personal things. They took the time to get to know him.
Continuity of clinicians and care	Maintaining clinician continuity over time and across health care transitions	Caregiver: I think he really liked… having the same nurse for when he had his infusions. And saw the same, you know—if his regular doctor wasn’t there, he saw the nurse practitioner, which he saw regular enough that he was able to be comfortable with them.
Compassionate care	Being treated with dignity, respect, compassion	Clinician: All I can think of is, you know, hold their hand, listen to what they’re saying. There are a lot of instances where you’re as powerless as they are... you do the best you can, provide empathy, compassion.
**Care and treatment**		
Cancer-directed therapy	Preferences about receipt of cancer-directed therapy	Patient: It’s also, at some point you hit the threshold where you just don’t gain anything. Sure you might be on chemotherapy at the end of your life, but what is it really getting you? There are diminished returns at some point. So, some people don’t care that your returns are diminished, some people do.
		Patient: Depending on how much worse my cancer would get, I would definitely be willing to compromise my quality of life, especially for a certain amount of time, to take a chance on another medication or drug that could be successful for me.
		Clinician: It’s all about what makes sense for that person.
Life-sustaining therapy	Preferences about life-sustaining measures, such as intensive care, ventilation, resuscitation	Patient: I guess I’d want to know what the benefit is of having all of that extra, if they know that you are dying anyways, you know? If it’s an attempt to make things most comfortable as possible, you know, then fine. But if it’s an attempt to just prolong life just for the sake of prolonging life for another like, week, you know—I don’t know.
		Patient: As aggressive as a treatment needs to be—that’s what I am going to do. I guess I take on a competitive, kind of a fighter mentality.
		Clinician: We kind of say sometimes—although we don’t like it—some patients do need to die in the intensive care unit. Some patients have to do that. They have to feel like they did absolutely everything possible. That doesn’t matter what I feel about that. It’s really what they feel.
Location of death	Preferred place of dying	Patient: Yeah, be out of the hospital.
		Caregiver: And that very day, as we were driving home, she said, “Mom if I have to die, I would like to die at home, but I’ll understand if I have to go to the hospital.” …So, it was at that point—in the ER—that I was like, “This is going to have to be in the hospital. I can’t do this.”
		Clinician: I think people are scared to die at home. They don’t understand what it will look like to die at home. They’re worried to burden their family… And I think for my patient, I think he thought that his suffering would be less if he died in a health care setting.
**Independence**		
Not burdening others	Not creating physical or emotional burdens on loved ones	Patient: But also what's really important to me is not being a burden on my loved ones.
Independence in medical care	Having independence with physical tasks of medical care and self-care	Clinician: He wanted to try to maintain his independence, but his disease made it nearly impossible to do that.

^a^ When needed to describe a range of perspectives, more than 1 quote may be included.

Each domain included relevant subdomains. For example, under attention to physical symptoms, participants prioritized attention to comfort and freedom from symptoms and suffering, but also the opportunity to maintain cognitive awareness when possible. The quality-of-life domain included global quality of life, but also attainment of life goals, legacy, and meaning; support of personal relationships; and the opportunity to maintain a sense of normalcy. Support of personal relationships included not just opportunity to be with loved ones and enjoy time together, but also support for the needs of those loved ones during and after the life of the AYA. Psychosocial and spiritual care included support in both domains, with acknowledgment that spiritual care includes support in dealing with existential challenges encountered by young people facing death.

For the domain of communication and decision-making, relevant subdomains included communication about prognosis, communication about what to expect at the end of life, timely communication early in the disease course, and opportunity for the AYA to hold their desired role in decision-making. In this domain, emphasis was placed on ensuring that AYAs have a say in the information they receive and when, as well as opportunity for their values for care to be heard and respected. In the related domain of relationships with clinicians, participants emphasized the importance of a strong therapeutic alliance with clinicians as well as continuity and compassion.

The domain of care and treatment included subdomains focused on use of cancer-directed therapy, use of life-sustaining therapy, and location of death. Although most participants emphasized a desire to avoid cancer therapy and intensive measures if they were unlikely to be effective, a minority of patients and family members expressed a desire to do everything possible to prolong life. Clinicians, too, noted that preferences are highly individual and that the best care for this population meets those individual preferences.

Finally, participants emphasized a need for independence in medical care when possible as well as freedom from burdening others.

Table 3 shows proposed quality indicators in each domain and subdomain and recommendations about whether new or existing indicators might best represent each.^40,41,42,43^ For example, attention to pain and symptoms is encompassed in a number of existing indicators for adults. However, we were unable to identify existing indicators that supported maintaining an experience of normalcy. We therefore proposed development of new indicators to support this priority.

**Table 3.** Quality Domains, Descriptions, Potential Indicators, and Recommendations for Development

Domain	Description	Potential quality indicators	Recommendation
**Attention to physical symptoms**			
Attention to comfort; freedom from symptoms and suffering	Attention to global comfort as well as specific symptoms, including pain, dyspnea, nausea, vomiting, fatigue, anxiety, depression, and constipation Patients may have different goals for symptom management, especially if some symptoms are more tolerable than others	Screening for symptoms; if symptom is present, intervention provided If intervention provided, reassessment of symptom occurs (within 24 h for inpatients, at next visit for outpatients) Patient report of whether goals for symptom control are met	Similar to domains in older adults Existing indicators may be appropriate^21,22,23,24,25^ May require new patient-reported indicator
Cognitive awareness	Opportunity to maintain cognitive awareness when possible	Patient report: clinicians asked about preference for level of wakefulness and respected that preference whenever possible Note that patients and family caregivers may have different preferences	Not typically addressed in existing quality indicators; may require new indicators
**Attention to quality of life**			
Global quality of life	Attention to quality of life, including recognition of domains important to patients, and maximization of quality of life whenever possible	Patient report on global quality of life Patient report: clinicians understood salient aspects of quality of life and supported whenever possible	Consider use of existing indicators of quality of life; end-of-life-specific domains for AYAs may require new indicators Likely requires new indicator to address clinician understanding of quality-of-life aspects
Attainment of life goals, legacy, and meaning	Opportunity to achieve life goals and milestones (eg, graduation, marriage) Experience of purpose and meaning in life, including legacy beyond life	Documentation of life goals and experiences as part of goals of care conversations Patient report: does care team understand what is most important to you to achieve or experience in your life?	May require new indicators
Personal relationships	Support of loved ones during AYA’s illness and after death Opportunity to maintain or deepen important relationships	Clinicians offer patients opportunity to discuss needs of family members Clinicians assess patient worries about their family members Clinicians know who are the important people in the patient’s life Clinicians help patients and family members communicate about issues that are important to them	May require new indicators due to enhanced importance or unique manifestations in AYAs
Maintaining a sense of normalcy	Opportunity to continue aspects of life that allow patient to feel normal (eg, work, school) Being seen as a person rather than a patient	Patient-clinician communication about what it means to feel “normal” and have a regular life Patient feels supported by clinician in maintaining normal life whenever possible Patient-clinician communication about identity and what is most important to patient	May require new indicator
**Psychosocial and spiritual care**			
Spiritual support	Support for spiritual and religious needs when desired Includes consideration of existential challenges of AYAs facing death	Patient was offered support of chaplain or spiritual careperson Team assessed and documented spiritual beliefs and religion Patient report: Care team addressed need for discussing effect of illness on sense of meaning and place in the world	Existing indicators may be appropriate^22,23,24^; consider augmenting with patient reports of issues with high relevance in AYAs
Global psychosocial support	Support for psychosocial dimensions of care, including patient depression and anxiety as well as support of family communication and emotional needs	Patient had at least one consult with a social worker or mental health professional Patient (family) report that psychosocial care met patient needs Patient (family) report that psychosocial care met needs for family communication and emotional support Assessment and management of anxiety and depression (above under symptoms)	Existing indicators may be appropriate^22^; consider addition of patient-reported indicators on family communication
**Communication and decision-making**			
Communication about prognosis	Discussions about prognosis take place, but in a way that is sensitive to individual needs, especially of younger patients Patients have opportunity to participate in or defer discussions about prognosis or delegate discussions to family members	Patient information needs assessed Patient received desired information about prognosis Documented discussion about prognosis or documentation that patient does not wish to discuss	May require AYA-specific indicators that consider the importance of patient involvement and choice in discussions
Communication about what to expect at the end of life	Information for patients and family members about what to expect as death comes closer, including physical changes, emotional needs, concrete and logistical information, and available support	Patient and family received the information they wanted about what to expect at the end of life	Existing indicators may be appropriate^40^
Timely communication	Patients (families) receive information about prognosis and what to expect at the end of life at the right time, early enough to allow for processing of information and planning	Patient (family) report that communication about prognosis and what to expect at the end of life occurred at the right time	Consider new indicators using patient (family) report
Holding desired role in decision-making (including receiving needed support and holding appropriate independence)	Patients are able to be involved in decision-making to the extent they wish, but also receive support when needed from clinicians and other important people in their lives	Support and guidance in decision-making from clinician Able to hold desired role in decision-making with clinician, with trusted others (parents, partners, others)	Consider indicators assessing role preferences^41^; enhanced importance or unique manifestations in AYAs
**Relationships with clinicians**			
Therapeutic alliance with clinicians	Experiencing a human bond and sense of connection with clinicians Seeing clinician as a source of support during illness Clinician allows patient to feel heard and understood	Therapeutic alliance	While not included in current quality indicators, existing instruments on therapeutic alliance may be appropriate^42^
Continuity of clinicians and care	Opportunity to see one’s own clinician as much as possible When health care transitions take place, having opportunity to maintain connection with continuity clinician or team	Continuity of care by clinician Transitions in health care settings	Consider indicators of continuity and transition used in adults^43^
Compassionate care	Care that respects dignity, delivered with respect, caring, compassion Clinicians treat patient as a whole person	Patient (family) reports of compassion (respect, dignity, caring)	Existing indicators may be appropriate^40,42^; may have enhanced importance or unique manifestations in AYAs
**Care and treatment**			
Cancer-directed therapy	Preferences are variable, with some patients wanting to pursue all possible options for as long as possible, and others only wanting to use cancer-directed therapy that has a reasonable prospect of benefit. Measures need to consider patient preferences, the extent to which patients are informed about treatment intent, and the extent to which clinicians are informed about patient goals	Patient (family) understanding of treatment intent Use of cancer therapy consistent with patient (family) values Patient-clinician (or patient-family-clinician) concordance on goals of cancer-directed therapy When cancer therapy is initiated, it is accompanied by goals of care discussion Patient report: patient feels that clinician understands personal goals for care and treatment Potential administrative and medical record measures: Documentation of treatment intent Documentation of goals of care at time of initiation of treatment Documentation of conversation about goals of care at time of initiation of treatment	Indicators need to be preference-based rather than focused on use and timing of chemotherapy
Life-sustaining therapy	Preferences are variable, with some patients wanting to pursue all possible options for as long as possible, and others only wanting to use life-sustaining therapy that has a reasonable prospect of benefit. Measures need to consider patient preferences, the extent to which patients are informed about prognosis, and the extent to which clinicians are informed about patient goals	Use of life-sustaining therapy consistent with patient (patient-family) values Patient-clinician (or patient-family-clinician) concordance on goals of care Decision about life-sustaining therapy should be accompanied by goals of care discussion Patient report: patient feels that clinician understands personal goals for care and treatment Potential administrative and medical record measures: Documentation of goals of care at time of initiation of life-sustaining therapy Documentation of conversation about prognosis and goals of care at time of initiation of life-sustaining therapy	Indicators need to be preference-based rather than focused on use and timing of life-sustaining measures
Location of death	Opportunity to die in preferred location; preferred location was highly variable and dependent on personal preferences, medical needs, and needs and wishes of family members	Death in place of one’s choosing Preferred place of death documented in medical record Patient and family feel that all efforts made to meet preferences around location of death Opportunity to be at home as long as comfort and support can be provided Also consider: When patient dies at home, hospice services are in place When patient dies in the hospital, palliative care services are in place	Indicators need to be preference-based rather than focused on home death and involvement of hospice
**Independence**			
Not burdening others	Desire for a care plan that considers burdens on others, especially family members	Patient report: Care team provided information about the level of care and assistance needed; caregiving was an important focus of the care plan	Not included in existing indicators; will require new patient-reported indicator
Independence in medical care	Patient is supported in being as independent as possible in medical care and self-care	Patient report: Patient was able to hold desired level of independence in medical care and self-care	May require new questions about independence in self-management

## Discussion

Interviews with AYAs, caregivers, and clinicians identified 7 distinct domains of end-of-life care quality for this population. In addition to existing domains, we identified new areas as well as unique manifestations of existing domains relevant to this young population. For example, we identified attainment of life goals and meaning as well as experiences of normalcy as key constructs of quality of life for AYAs. Adolescents and young adults also identified a need for independence as a core domain missed in existing quality indicators.

We also found that some indicators, especially aspects of care delivery such as end-of-life cancer therapy or location of death, were dependent on the preferences of individual patients. Many participants, across all participant types and AYA ages, agreed that late-life chemotherapy is undesirable if there is little prospect of benefit. However, others felt that trying to prolong life, even at the cost of quality of life, can be significant for young people whose lives are being cut short, as well as to patients who may have children or other loved ones for whom any additional time is worthwhile. Thus, all participants generally agreed that optimal care and treatment at the end of life for AYAs is not universal, but rather what is right for the individual patient.

The need for sensitivity to preferences was also emphasized in indicators related to communication and decision-making. Although many AYAs want to hear about prognosis, for example, some may prefer to defer these conversations or delegate them to other trusted individuals. For these indicators, we emphasize that patients need a say in how, when, and with whom these conversations take place.

Because many indicators are highly individualized and focused on the patient experience, many will require patient and/or caregiver report. This need poses challenges to large-scale quality assessment. Where possible, we have proposed potential administrative or medical record–based indicators to facilitate more widespread data collection. However, sole reliance on administrative indicators will miss many of the constructs that are salient to this young population. We therefore advocate for use of a patient or caregiver report when needed and possible. These new indicators require further development and validation across a diverse population.

Assessment of AYA end-of-life care quality will require careful consideration of implementation methods. Because patient report is of value, prospective identification of appropriate populations will be required, and we currently lack a definition of which patients might be most appropriate for such assessment and when. We included patients with stage IV or recurrent disease in this study, and although some had the prospect of a cure, they were able to discuss end-of-life issues because they were already thinking about these issues. Thus, a relatively inclusive population of patients with a limited prognosis from the time of diagnosis may be appropriate, despite our own use of the term *end of life*; in fact, some areas may be salient to all AYAs with cancer. In addition, processes around data collection and reporting, including whether findings should be presented in the aggregate or available at the individual level for review by clinicians, patients, and caregivers, will need to be defined. These questions offer the opportunity to define health systems that are responsive to the needs of AYAs with advanced cancer and their families.

### Limitations

This study has some limitations. Racial and ethnic diversity was limited, and no Asian or Spanish-speaking patients or caregivers participated. The preferences of a diverse population need continued focus. In addition, by requesting permission from clinicians and screening with the readiness assessment, we may have identified patients who were more open to conversations about end-of-life care and whose values reflected those preferences. Notably, many patients declined participation. However, inclusion of family caregivers and clinicians was used to ensure a diversity of experiences, and clinicians were specifically probed to consider recent patients without respect to the choices they made for care. Our next step, a Delphi process (ie, a structured communication technique to help a group arrive at a decision) with patients, caregivers, and clinicians to prioritize identified indicators, will allow us to obtain broader input on our findings with renewed efforts to include a diverse population. We interviewed few patients in the youngest age range, in part due to challenges of in-person interviews during the COVID-19 pandemic. We dealt with this by enrolling more caregivers of adolescents and ensuring inclusion of pediatric clinicians. The content from interviews with younger patients was consistent with that from older patients. However, the needs of younger AYAs require ongoing attention and emphasis. Although no interviewers were known to the participants, 1 interviewer was a pediatric oncologist, raising concerns about reflexivity. We worked to mitigate this concern by involving a patient advocate at each step from interview development to analysis.

## Conclusions

Despite recommendations from the National Academy of Medicine for inclusion of stakeholder voices in the development of quality indicators, inclusion of patient and caregiver perspectives remains an underused aspect of quality indicator development.^32^ In addition to revealing important quality domains for AYAs, some of the domains we identified in this qualitative study may have relevance to older adults as well. These domains may have not been previously identified owing to more limited use of patient perspectives in quality measure development. Although these topics were difficult for young people and their family members to discuss, many strongly desired to express what was most important to them and sought care that would support those personal values. Responsive health care professionals and systems can honor these values by systematically measuring and reporting salient areas to promote high-quality care for young people at the end of life.

## Notes

Supplement. **eTable.** Sample Patient Interview Questions
