# Supplementary material for: Patient, Family, and Clinician Perspectives on End-of-Life Care Quality Domains and Candidate Indicators for Adolescents and Young Adults With Cancer
Source: JAMA Netw Open. 2021 Aug 23;4(8):e2121888. doi: 10.1001/jamanetworkopen.2021.21888 (PMC8383130; doi:10.1001/jamanetworkopen.2021.21888)
Supplement: Supplement. — eTable. Sample Patient Interview Questions [file jamanetwopen-e2121888-s001.pdf]

Supplementary Online Content

Mack JW, Fisher L, Kushi L, et al. Patient, family, and clinician perspectives on end-of-life care quality domains and candidate indicators for adolescents and young adults with cancer. *JAMA Netw Open*. 2021;4(8):e2121888. doi:10.1001/jamanetworkopen.2021.21888

eTable. Sample Patient Interview Questions

This supplementary material has been provided by the authors to give readers additional information about their work.

© 2021 Mack JW et al. *JAMA Network Open.*

eTable. Sample Patient Interview Questions

Introductory questions for all patients	I would like to ask you some questions about what you think is most important to people your age with cancer if treatment is no longer working (and cure is not possible). That may not be the situation you are in, but we think as a young person with cancer, you can help us understand your perspective and what matters to young people in that situation.<br><br>I'd like to learn a little bit about your illness. Can you tell me what you believe is ahead for you in your illness? [*Probing questions if needed:* what do you think might happen to you because of your illness? How do you think your illness can affect you in your future? In what ways might your illness affect your future plans or goals?]
Follow-up questions for patients who acknowledge a poor prognosis	You just told me that you think about *[use patient's words.]* Some patients start to make plans for the care they would or would not like to receive if treatment doesn't work well. Is this something you have thought about?<br><br>*If **YES**, patient **HAS** thought about care plans:* What kinds of things have you thought about? Have you discussed your thoughts with anyone else? If so, who?<br><br>*If **NO**, patient **HAS NOT** thought about care plans:* As you think about your life right now, what things are most important to you? Are there things that are especially important to achieve or accomplish? Are there things that you especially value having in your life?<br><br>What about when you think about your medical care? What is most important to you in your care right now?<br><br>What about things that you would like to be sure do not happen to you, either in your life or in your medical care?<br><br>Now I am going to ask you an especially difficult question. You can tell me you want to move on and not answer it if you want. Okay? Most of us have "what if" conversations that come to our mind. We tend to have them when we are alone, or trying to sleep. If you were faced with a time when it seemed that you could die of your illness, can you imagine what might be most important to you at that time? Tell me about that. Can you imagine anything you might not want to happen to you? What else has gone through your mind?<br><br>These are hard things to talk about, but I'm grateful that you are sharing your thoughts. Are you okay for a few more questions?<br><br>Okay. Now I would like to talk about what we know about the experiences of young people with cancer who can't be cured, and who eventually die of their cancer. We know that, in their last few weeks of life, most of them experience a lot of medical care, like chemotherapy, time in the hospital or emergency room, and time in the intensive care unit. What do you think about that? Does that sound like the kind of care you would want, the kind of care you wouldn't want, or would it depend? Why?<br><br>We also know that many young people die in the hospital, although some die at

© 2021 Mack JW et al. *JAMA Network Open.*

	home. Can you imagine what you would want? Why?<br><br>Are there any ways you would want to be sure that your life has had meaning?<br><br>We have talked about different priorities that you have, and also different priorities that other young people have. As you think about the many different things that are important to you, what do you think is most important for doctors and nurses to understand about you? If you could tell your doctor or nurse anything about what matters to you most, what would you tell him/her?<br><br>Is there anything else you would like for me to know about the things we have talked about? Are there other things that you would like to express about what might be important to people your age if they get to a point when treatment is no longer working?<br><br>Thank you for talking with me about these things. I know these are hard topics but I have learned so much from your perspective.
Follow-up questions for patients who do not acknowledge a poor prognosis	As you think about your life in general, what parts are most important to you?<br><br>What is most important to you in your care right now?<br><br>What about things that you would like to be sure do not happen to you, either in your life or in your medical care?<br><br>Now I would like for you to imagine something difficult- I would like for you to imagine that treatment was no longer working for you. I am glad that you aren't in this situation, but I am hoping you can help me understand what might be important to you if you were.<br><br>What parts of your life do you imagine might be most important to you if treatment were no longer working?<br><br>Some patients start to make plans for the care they would like to receive if treatment doesn't work well. Can you imagine what kinds of things might be important to you about your medical care if you were ever in that situation?<br><br>Now I am going to ask you an especially difficult question. You can tell me you want to move on and not answer it if you want. Okay? Most of us have "what if" conversations that come to our mind. We tend to have them when we are alone, or trying to sleep. If you were faced with a time when it seemed that you could die of your illness, can you imagine for me what might be most important to you at that time? Tell me about that. Can you imagine anything you might not want to happen to you? What else has gone through your mind?<br><br>These are hard things to talk about, but I'm grateful that you are sharing your thoughts. Are you okay for a few more questions?<br><br>Okay. Now I would like to talk about what we know about the experiences of young people with cancer who can't be cured, and who eventually die of their cancer. We know that, in their last few weeks of life, most of them experience a lot of medical care, like chemotherapy, time in the hospital or emergency room, and time in the intensive care unit. What do you think about that? Does that sound like the kind of care you would want, the kind of care you wouldn't want,

© 2021 Mack JW et al. *JAMA Network Open.*

	or would it depend? Why?<br><br>We also know that many young people die in the hospital, although some die at home. Can you imagine what you would want? Why?<br><br>Are there any ways you would want to be sure that your life has had meaning?<br><br>We have talked about different priorities that you have, and also different priorities that other young people have. As you think about the many different things that are important to you, what do you think is most important for doctors and nurses to understand about you? If you could tell your doctor or nurse anything about what matters to you most, what would you tell him/her?<br><br>Is there anything else you would like for me to know about the things we have talked about? Are there other things that you would like to express about what might be important to people your age if they get to a point when treatment is no longer working?<br><br>Thank you for talking with me about these things. I know these are hard topics but I have learned so much from your perspective.

© 2021 Mack JW et al. *JAMA Network Open.*
